# Supplementary material for: Shorter Grazing Time and Supplementation Are Beneficial for Gastrointestinal Tract Development and Carcass Traits of Growing Lambs
Source: Animals (Basel). 2022 Mar 30;12(7):878. doi: 10.3390/ani12070878 (PMC8996875; doi:10.3390/ani12070878)
Supplement: Supplementary file 1 [file animals-12-00878-s001.zip › Table S1.pdf]

Table S1.Amounts of concentrate, grass hay and pasture consumed by lambs and herbage allowance of pasture in Inner Mongolia, China

Item	Treatment[1]			
	2H	4H	8H	12H
Concentrate[2] (kg/day)	0.40	0.27	0.23	0.12
Grass hay (kg/day)	0.34	0.25	0.13	0
Pasture (kg DM/day)	0.62	0.89	0.99	1.24
Total dry matter intake (kg/day)	1.36	1.41	1.35	1.36
Crude protein intake (g DM/day)	177.87	184.94	180.95	180.63
Metabolisable energy intake (MJ/day)	13.83	14.08	13.40	13.13
Neutral detergent fibre intake (g DM/day)	645.41	761.73	749.94	835.85
Herbage allowance (kg DM/ha)	992.00	952.87	951.03	932.41

[1]Treatments: 2H = 2 h access to pasture; 4H = 4 h access to pasture; 8H = 8 h access to pasture; 12H = 12 h access to pasture.

[2]The concentrate consisted of 63% chopped maize, 10% wheat bran, 10% soybean meal, 8% cottonseed meal, 8% rapeseed meal and 1% premix. The premix contained (per kg) 10500 IU vitamin A, 2110 IU vitamin D3, 43 mg vitamin E, 40 mg Mn, 32 mg Fe, 95 mg Zn and 16 mg Cu.
